# Occluded Middle Cerebral Artery Vascular Stump Mimicking Aneurysm: Case Report and Review of Literature

**DOI:** 10.12669/pjms.293.2839

**Published:** 2013

**Authors:** Jinlu Yu, Yang Zhang, Honglei Wang

**Affiliations:** 1 Jinlu Yu, MD, Departments of Neurosurgery, The First Affiliated Hospital of Jilin University, Changchun, Jilin, China.; 2 Yang Zhang, MD, Departments of Neurosurgery, The First Affiliated Hospital of Jilin University, Changchun, Jilin, China.; 3 Honglei Wang, MD, Departments of Neurosurgery, The First Affiliated Hospital of Jilin University, Changchun, Jilin, China.

**Keywords:** MCA bifurcation, Arterial occlusion, Aneurysm

## Abstract

The stump of an occluded middle cerebral artery (MCA) is a rare but important aneurysm mimic. A 57-year-old male was admitted due to recurrent onset of transient ischemic attack. Computed tomography angiography (CTA) and digital subtraction angiography (DSA) showed a total obstruction in the right MCA with moyamoya phenomenon at distal trunks and a protruding lesion in the left MCA bifurcation. The patient was diagnosed with left MCA bifurcation aneurysm. Intraoperatively, the lesion was found to be an occluded right MCA stump. Encephalomyoarteriosynangiosis was performed, and the patient remained symptom-free at the 6-month follow-up. The possibility of a vascular stump should be considered when an aneurismal lesion is present at the MCA bifurcation with moyamoya phenomenon at distal trunks.

## INTRODUCTION

Intracranial vascular lesions, such as a vascular loop, infundibulum, and stump of an occluded vessel, are sometimes misdiagnosed as aneurysms during imaging examinations.^1^ It is difficult to differentiate such lesions from aneurysms on the basis of imaging findings.^2^^,^^3^ Recognition of aneurysm mimics is crucial to triage patients for the appropriate treatment. Previous reports have found that most vascular stumps mimicking aneurysms are observed in the posterior circulation.^1^^-^^4^ Here, we report a unique case of an occluded middle cerebral artery (MCA) stump mimicking an MCA bifurcation aneurysm.

## CASE REPORT

A 57-year-old man was admitted to our department due to recurrent onset of transient ischemic attacks during the past week. The patient presented with right-sided weakness and aphasia during each attack. The symptom lasted for approximately 5 minutes and disappeared completely after each attack. Brain computed tomography (CT) scan and magnetic resonance imaging (MRI) were performed immediately after admission and did not show any ischemic changes (Fig.1A). A CT angiography (CTA) was performed and showed total occlusion at the proximal segment of the right MCA. A protruding lesion of 2.1 mm × 3.8 mm was seen in the left MCA bifurcation with distal moyamoya phenomenon (Fig.1B). The patient was diagnosed with left MCA bifurcation aneurysm on the basis of the CTA findings.

Digital subtraction angiography (DSA) was performed and showed occlusion of the right MCA and saccular out-pouching of the left MCA bifurcation. The three-dimensional reconstruction angiogram was consistent with an aneurismal lesion. A moyamoya lesion was noticed at the distal segments beyond the site of occlusion (Fig.1C). The patient was diagnosed with MCA bifurcation aneurysm and transient ischemic attack.

We planned to perform clipping of the MCA bifurcation aneurysm and encephalomyoarteriosynangiosis (EMAS). Intraoperatively, the lesion was found to be an occluded right MCA stump (Fig.1D). The patient was treated with 100 mg/day of aspirin and was discharged at one week after surgery. The patient did not suffer from transient ischemic attack or seizures for six months after surgery.

## DISCUSSION

Most residual stumps are seen in the posterior circulation, in 1992, Kalia et al. reported a case of thrombosed, fenestrated basilar artery mimicking an aneurysm of the vertebrobasilar junction.^5^ Since then, stumps have been reported in several locations.^2^^,^^3^^,^^6^ In early studies, stumps in the posterior circulation were often misinterpreted by neurosurgeons as aneurysms on the basis of the imaging findings. Recently technical advancements have helped with the diagnosis of vascular stumps, and the lesions are increasingly being recognized by neurosurgeons before surgery. In 2001, Komiyama et al. reported a case of vertebral artery occlusion in which the diagnosis was made on the basis of DSA findings before surgery.^2^

**Fig.1A:**
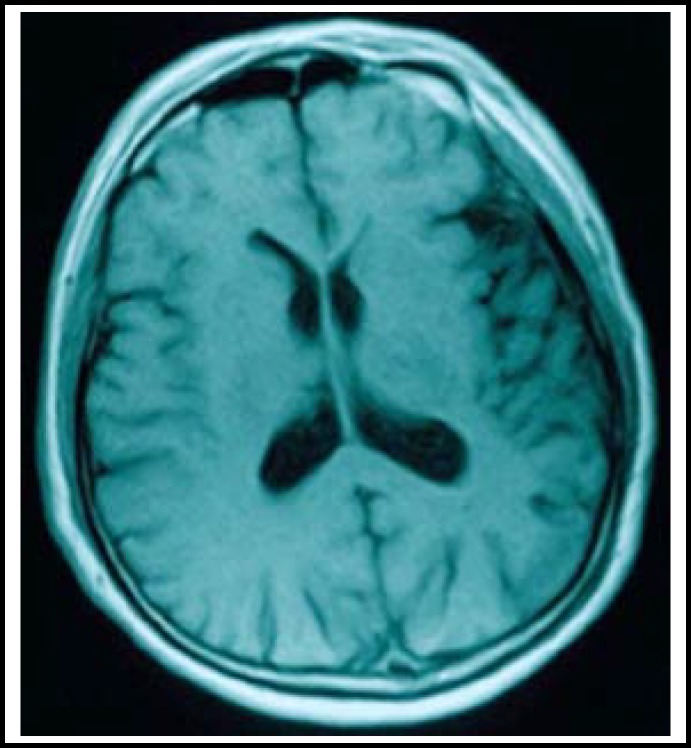
Admission MRI of the patient showed that there were no abnormalities in T1-weighted imaging

**Fig.1B:**
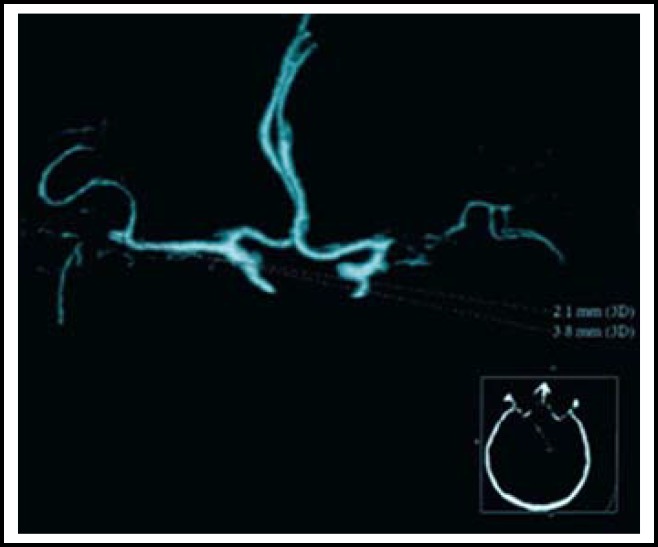
Admission CTA of the patient shows right MCA M1 occlusion and left MCA bifurcation stump, the stump measured 2.1 mm × 3.8 mm on CTA volume-rendered image

**Fig.1C:**
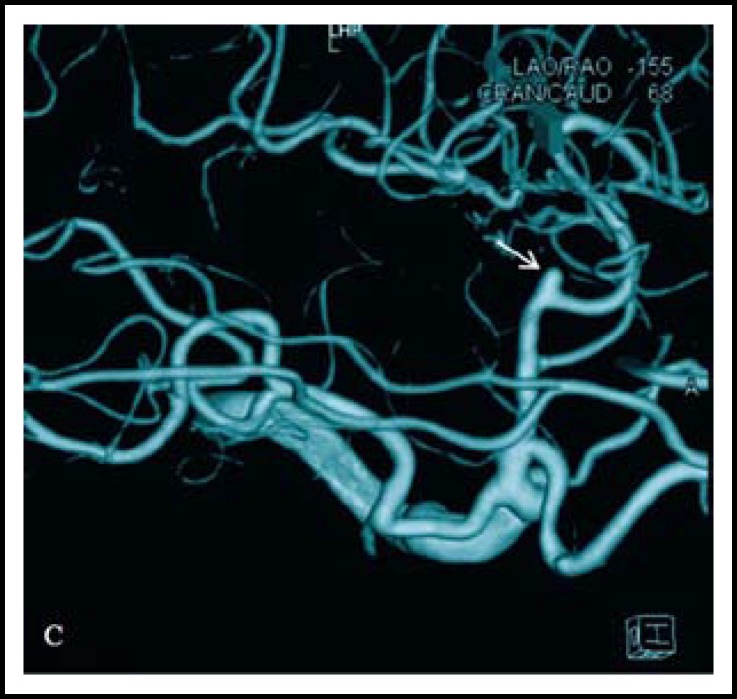
DSA shows right MCA M1 occlusion and moyamoya phenomenon around the site of occlusion. The vascular stump (white arrow) was clearly visualized on 3D DSA reconstruction image

**Fig.1D:**
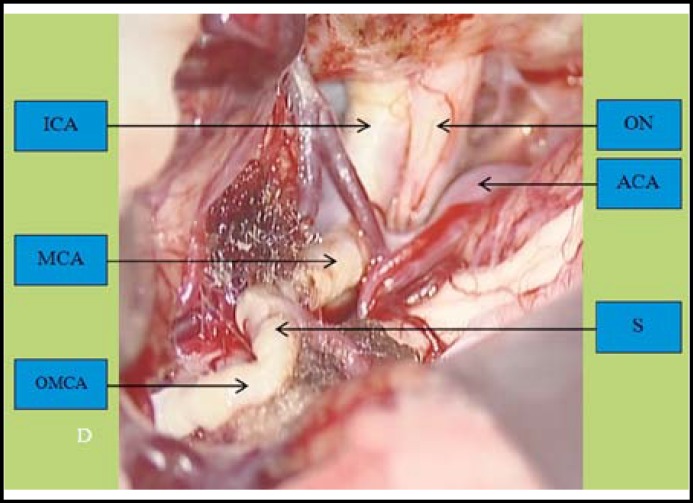
Intraoperative view. ICA, internal carotid artery; ON, optic nerve; ACA, anterior cerebral artery; MCA, M1 segment of the middle cerebral artery; S, Stump; OMCA, occluded middle cerebral artery segment

As per our knowledge, an MCA vascular stump mimicking an MCA bifurcation aneurysm is extremely rare. Several cases of MCA vascular stump have been reported.^1^^,^^4^^,^^7^ Previous studies have suggested that the MCA M2 segment may split into 2 or 3 main trunks.^8^ In the case of a trifurcation, the stump of an occluded M2 segment may be easily recognized as an aneurysm located at the MCA bifurcation on the basis of the imaging findings. In 2008, Park et al^1^. reported two cases of M2 segment stumps in patients with MCA trifurcation. In these cases, the DSA findings identified the occluded stumps as aneurysms. The vascular stumps were diagnosed during surgical clipping of the aneurysm.

Vascular stumps could be differentiated from aneurysms based on anatomical features. A vascular stump should be considered in patients with unilateral MCA bifurcation aneurysm, in which the contralateral MCA usually divides into three branches. In case of an MCA M2 segment occlusion, vascular ischemic changes may occur in the corresponding vascular territory.^7^^,^^9^ In 2008, Pearl et al^7^. successfully diagnosed a case of MCA M2 stump from the DSA findings. The occluded artery was recanalized on follow-up DSA examination.

We report a unique case of MCA M2 vascular stump. Because the right MCA was occluded, we could not compare the vascular anatomy of both sides from the imaging findings. A diagnosis of MCA bifurcation aneurysm was made on the basis of the DSA findings. The MCA M1 branch was divided into two trunks. The inferior trunk was absent on the CTA and DSA, which may have suggested a possible vascular stump rather than an aneurysm. Interestingly, the moyamoya phenomenon was noticed in the distal segments of the occluded vessel. The moyamoya phenomenon also was reported by Park et al. in 2008. Aneurysm can occur in patients with moyamoya disease due to hemodynamic changes.^10^ In our case, the unusual location of the moyamoya phenomenon may have provided a clue to the diagnosis of a vascular stump.

We made the diagnosis of vascular stump during surgery. Collateral circulation distal to the occluded site was found. In the case of a vascular stump, previous studies have suggested that clipping should not be performed.^1^^,^^4^ In our case, the distal location of the moyamoya phenomenon relative to the occlusion site indicated compensation of the collaterals. However, the patient had symptoms of transient ischemia, which suggested that the compensation was inadequate to maintain sufficient blood flow. Therefore, EMAS was performed to reestablish blood flow. The patient remained symptom-free at the 6-month follow-up.

## CONCLUSIONS

The possibility of a vascular stump should be considered in patients with MCA bifurcation aneurysms. Moyamoya phenomenon distal to the site of occlusion also may provide a clue to the diagnosis of a vascular stump rather than aneurysm.

## Authors Contributions

Jinlu Yu: Contributed in manuscript writing.

Yang Zhang: Was involved in clinical management of the patient. 

Honglei Wang: Performed the operation and reviewed the manuscript for final publication.
